# A retrospective cohort study comparing periodontal regeneration using fibroblast growth factor‐2 versus autologous bone graft

**DOI:** 10.1002/jper.70060

**Published:** 2026-01-19

**Authors:** Toshiki Matsumoto, Shin Nakamura, Yuki Ito‐Shinoda, Mai Sakamoto, Takayuki Ishii, Yasuki Nonomura, Hidetaka Ideguchi, Keisuke Okubo, Kazu Takeuchi‐Hatanaka, Kazuhiro Omori, Tadashi Yamamoto, Shogo Takashiba

**Affiliations:** ^1^ Department of Pathophysiology–Periodontal Science Graduate School of Medicine, Dentistry and Pharmaceutical Sciences Okayama University Okayama Japan; ^2^ Department of Periodontics and Endodontics, Division of Dentistry Okayama University Hospital Okayama Japan; ^3^ The Center for Graduate Medical Education (Dental Division) Okayama University Hospital Okayama Japan

**Keywords:** autologous bone graft, fibroblast growth factor‐2, periodontal pocket, periodontal regeneration, periodontitis, vertical bone defect

## Abstract

**Background:**

Fibroblast growth factor‐2 (FGF‐2) is a novel agent utilized in periodontal regeneration therapy. However, its clinical efficacy compared with autologous bone graft (ABG), a long‐established treatment, remains unclear. This study aimed to compare the clinical outcomes of FGF‐2 and ABG and to assess the impact of patient background factors on outcomes when using FGF‐2.

**Methods:**

We collected the subjects from January 2013 to September 2023. Clinical outcomes included the vertical bone defect improvement rate (VBDIR) and the probing pocket depth improvement (PPDI). Clinical outcomes between the two groups were compared using analysis of covariance (ANCOVA), adjusting for age, sex, smoking history, and hypertension. Additionally, a multilevel linear analysis was performed to assess factors influencing outcomes in FGF‐2.

**Results:**

A total of 180 sites from 141 patients (FGF‐2: 150 sites; ABG: 30 sites) were evaluated. Both VBDIR and PPDI significantly improved postoperatively in both groups. There were no significant differences in clinical outcomes between FGF‐2 and ABG. In FGF‐2, smoking history was positively associated, while the preoperative bone defect angle (BDA) was negatively associated with clinical outcomes.

**Conclusions:**

FGF‐2 might exhibit clinical outcomes comparable to those of ABG, suggesting it is a clinically viable alternative for vertical bone defects. When using FGF‐2, patient‐specific factors such as smoking history and preoperative BDA should be considered carefully.

**The name in the trial registry:**

A survey of clinical practice and evaluation of treatment outcomes of periodontal regenerative therapy using REGROTH at Okayama University Hospital

**Plain language summary:**

This retrospective study compared the clinical outcomes of fibroblast growth factor‐2 (FGF‐2), a novel therapeutic agent, with autologous bone graft (ABG), a long‐established treatment. A total of 180 sites from 141 patients (150 FGF‐2 sites, 30 ABG sites) were evaluated based on the vertical bone defect improvement rate (VBDIR) and the probing pocket depth improvement (PPDI). Using analysis of covariance (ANCOVA), adjusted for patient background factors such as age, sex, smoking history, and hypertension, no significant differences in clinical outcomes were observed between the two treatment groups. Further multilevel linear analysis focusing on the FGF‐2 group revealed that smoking history was positively associated, while preoperative bone defect angle (BDA) was negatively associated with clinical outcomes. These findings suggest that FGF‐2 may offer clinical benefits comparable to ABG in treating vertical bone defects while having the added advantage of being less invasive. However, when using FGF‐2, dentists should consider individual patient factors such as smoking habits and defect morphology, as these may influence treatment outcomes.

## 1 INTRODUCTION

The progression of periodontal disease leads to the destruction of periodontal tissue, ultimately resulting in tooth loss and a reduced quality of life.^1^ In periodontal disease, dysbiosis of the subgingival microbiome–dominated by inflammophilic gram‐negative anaerobes–drives immune‐inflammatory responses that cause irreversible destruction of the tooth‐supporting tissues. Although the primary treatment for periodontal disease involves the mechanical removal of dysbiotic biofilm and inflammation, achieving true periodontal regeneration remains a clinical challenge. This is largely due to the rapid proliferation of epithelial tissue into the alveolar bone defect. Ideal periodontal regeneration requires minimal epithelial attachment, the formation of new cementum enclosing collagen fibers, and the formation of new bone.^2^ Periodontal regenerative therapy aims to restore lost structures, fill osseous defects, and ensure long‐term stability, rather than relying on the presumed weakness of long junctional epithelium.^3^ Therefore, various regenerative therapies have been developed to restore both the morphology and function of the damaged periodontal tissue, including bone grafts, guided tissue regeneration (GTR), the application of enamel matrix derivatives (EMD), and the use of growth factors.^4^, ^5^, ^6^, ^7^, ^8^

Among them, recombinant human basic fibroblast growth factor‐2 (FGF‐2) was introduced as the world's first approved periodontal tissue regenerative medicine and became covered by health insurance in Japan in 2016. FGF‐2 exhibits potent angiogenic properties and promotes the proliferation of undifferentiated mesenchymal stem cells, thereby facilitating the formation of new alveolar bone, periodontal ligament, and cementum.^9^, ^10^, ^11^, ^12^, ^13^ A randomized controlled trial in patients with chronic periodontitis demonstrated that FGF‐2 significantly promoted alveolar bone regeneration and exhibited comparable efficacy to EMD.^14^

Meanwhile, bone graft has the longest history in periodontal regeneration therapies, utilizing various materials such as autologous, allogeneic bone, xenogeneic, and artificial bone. Autologous bone graft (ABG)^15^ involves harvesting cortical bone from the patient's own alveolar bone, chin, mandibular ramus, or ilium for use as a regenerative material.^16^ ABG offers key advantages, including a low risk of immune reaction and the retention of osteo‐inductive, osteo‐conductive, and osteogenic properties.^17^, ^18^ Because of its properties and the absence of immunological reactions, autologous bone grafts have been considered as the “gold standard” and most effective material in bone regeneration procedures.^19^, ^20^ However, the limitations of ABG have been reported to include restricted donor sites and possible harvesting morbidity, reports of unpredictable resorption, and limited available bone volume for intraoral bone grafts,^21^ in addition to its main limitation of the invasiveness of the harvesting procedure and the restricted quantity of bone that can be obtained, making it less suitable for treating large bone defects. In contrast, allogeneic and xenogeneic bone grafts allow for unlimited supply, but their osteo‐inductive capacity and biocompatibility are generally inferior to those of autogenous bone.^22^ Similarly, synthetic materials such as hydroxyapatite and β‐tricalcium phosphate^23^, ^24^ have been reported to exhibit lower regenerative potential compared with ABG.^22^ These facts suggest that ABG is a well‐established and somewhat reliable regenerative material.

While the effectiveness of ABG and FGF‐2 in periodontal regeneration therapy has been demonstrated, few studies, including one retrospective study^25^ and one randomized control study,^26^ have directly compared the two modalities. Therefore, this study aimed to evaluate the regenerative efficacy of FGF‐2 in comparison with ABG, which has a long‐standing clinical track record. Additionally, we investigated the association between clinical outcomes and patient background factors–including age, sex, smoking history, and systemic conditions–with a particular focus on periodontal regeneration therapy using FGF‐2.

## 2 MATERIALS AND METHODS

### 2.1 Study design

This retrospective cohort study targeted patients who received periodontal tissue regeneration therapy at the Department of Periodontics and Endodontics, Division of Dentistry, Okayama University Hospital, during the period from January 2013 to September 2023. Participants were selected based on the following inclusion criteria: (1) patients diagnosed with periodontal disease, adequate oral hygiene management, and who had completed initial preparation (IP), including the removal of traumatic occlusion and inappropriate restorations; (2) patients aged 20 years or older; (3) Asian ethnicity; (4) sites with residual probing pocket depth (PPD) greater than 4 mm after IP; and (5) vertical bone defects greater than 3 mm as observed dental radiographs.

Exclusion criteria were as follows: (1) teeth from patients with osteolytic diseases such as rheumatoid arthritis, osteoporosis, or bone metastasis from cancer; (2) teeth presenting with furcation involvement; (3) teeth with endodontic‐periodontal lesions; (4) teeth treated with materials other than FGF‐2 or ABG, or teeth treated with FGF‐2 and ABG in combination; (5) teeth lacking clinical or radiographic follow‐up data within 2 years before and after surgery; and (6) teeth treated by dentists who performed only FGF‐2 or ABG therapy exclusively. Sample size calculation was not performed because this study was designed as a retrospective cohort study based on existing clinical records. Accordingly, the sample size was determined by the number of patients who met the eligibility criteria during the study period.

This study was approved by the Okayama University Ethics Committee (approval number: 2110‐018) and conducted in accordance with the Declaration of Helsinki (1975), as revised in 2008 and 2013. Information disclosure was carried out using an ethics committee‐approved document, and data were corrected from medical records after notifying patients through an opt‐out process. All data were anonymized to prevent the identification of individuals prior to analysis.

### 2.2 Clinical and radiographic analysis

#### 2.2.1 Clinical examination

Probing pocket depth was assessed by board‐certified dentists of the Japanese Society of Periodontology.

The PPD was measured at six sites on each tooth using a periodontal probe (YDM, Tokyo, Japan). PPD was defined as the distance from the gingival margin to the base of the gingival sulcus. The postoperative improvement in the PPD was evaluated as the PPD improvement (PPDI, mm), calculated relative to the preoperative baseline.

#### 2.2.2 Radiographic evaluation

**FIGURE 1:**
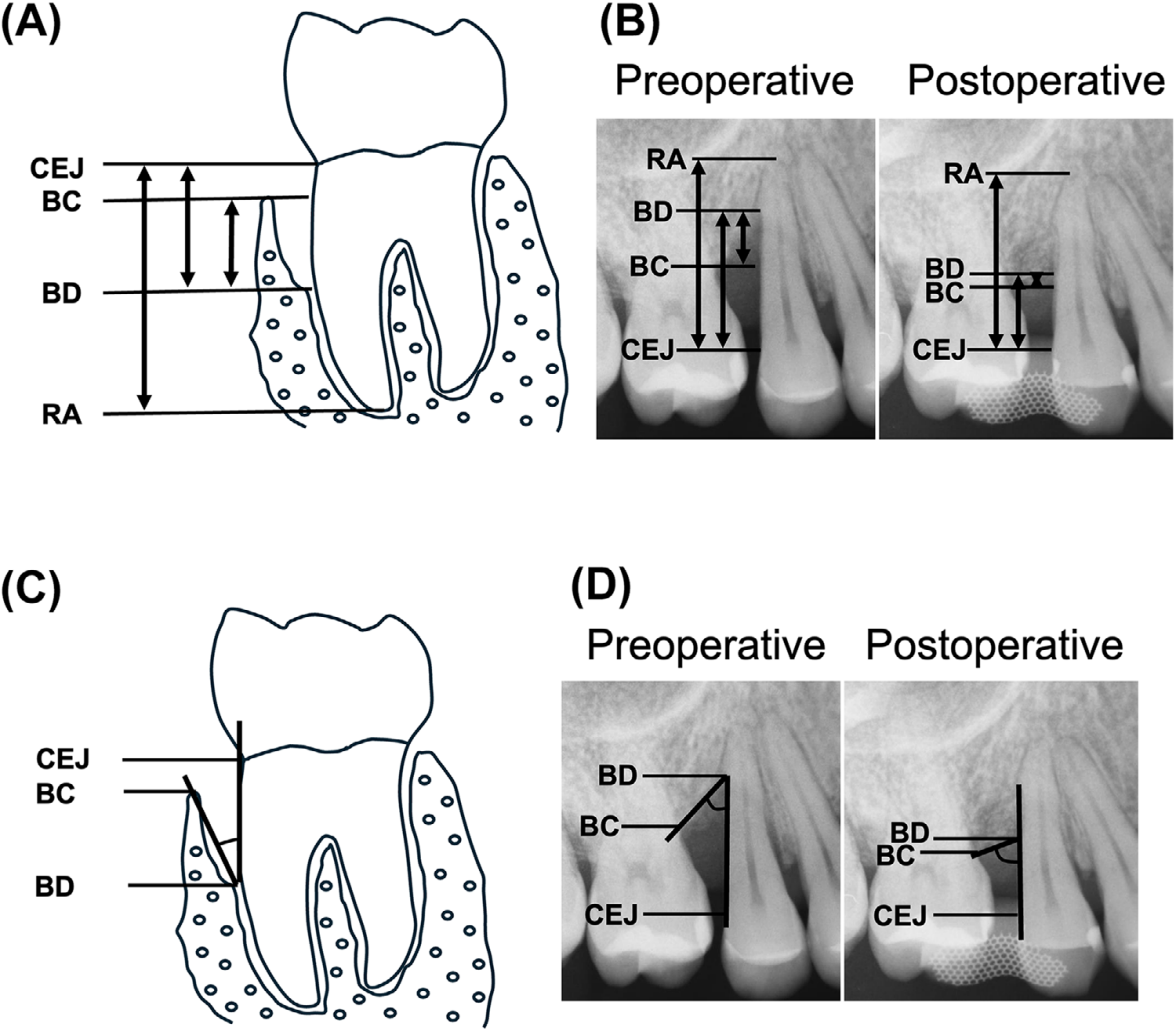
Measurement methods for vertical bone defect improvement ratio (VBDIR) and bone defect angle (BDA). (A and B) CEJ (cemento‐enamel junction), RA (root apex), BD (bottom of the intrabony defect), and BC (alveolar bone crest). On pre‐ and postoperative dental radiographs, the lengths of CEJ–RA, CEJ–BD, and BC–BD were measured to calculate the VBDIR. A schematic diagram indicating the measurement sites and representative radiographs before and after surgery are shown. (C and D) The angle formed between CEJ–BD and BC–BD was measured as the bone defect angle. The angles between CEJ–BD and BC–BD were measured. A schematic diagram indicating the measurement sites and representative radiographs before and after surgery are shown.

The vertical bone defect improvement ratio (VBDIR) was assessed using dental x‐ray images taken prior to surgery and at follow‐up visits between 9 months and 2 years postoperatively. Radiographic images were captured using a parallel technique with digital radiographic equipment (maxiX Type2, MORITA, Tokyo, Japan). All images were printed, and radiographic evaluations were conducted independently and in a blinded manner by four calibrated examiners (T.I., M.S., Y.N., T.M.). The calibration was performed using a sample radiograph prior to the evaluations. To ensure reproducibility in the measurement, we adapted the method described by Kojima et al.^26^ A correction factor (CF) was calculated based on the distance from the cemento‐enamel junction (CEJ) to the root apex (RA). The position of the bottom of the intrabony defect (BD) was measured preoperatively (PreO) and postoperatively (PostO). Using the distance from the top of the alveolar bone crest (BC) to BD at baseline, the VBDIR (%) was calculated using the following formula (Figure 1A and B):

$$\mathrm{CF}=\mathrm{CEJ}-\mathrm{RA}\left(\mathrm{PreO}\right)/\mathrm{CEJ}-\mathrm{RA}\left(\mathrm{PostO}\right)$$

$$\begin{array}{ccc} \mathrm{VBDIR} & = & \left(\mathrm{CEJ}-\mathrm{BD}\left(\mathrm{PreO}\right)-\mathrm{CEJ}-\mathrm{BD}\left(\mathrm{PostO}\right)\right)\\ & & \times \;\mathrm{CF}/(\mathrm{BC}-\mathrm{BD})\times 100 \end{array}$$

Additionally, the bone defect angle (BDA) was calculated using dental x‐ray images taken with the parallel technique before surgery and between 9 months and 2 years postoperatively. The BA was determined by measuring the angle formed between the line connecting the CEJ on the proximal root surface to BD and the line connecting the top of BC of the defect to BD (Figure 1C and D).

### 2.3 Surgical procedure

Under local anesthesia, a full‐thickness flap was created in all regenerative procedures. To preserve the interdental papilla, either the Simplified Papilla Preservation Technique or the Modified Papilla Preservation Technique was employed. Thorough debridement and root planing were conducted using an ultrasonic scaler (Varios G1, NSK, Tochigi, Japan) and hand instruments (Hu‐Friedy, Chicago, IL, USA). FGF‐2 (Regroth, Kaken Pharmaceutical Co., Tokyo, Japan) was prepared according to the manufacturer's instructions and injected into the bone defect. The formulation (0.3% rhFGF‐2 in HPC gel, 0.6 mL prefilled syringe) was applied to completely fill the intrabony defect, with the volume adjusted to defect size but standardized as the amount sufficient to fill the defect. For ABG, autogenous bone was collected from the marginal bone surrounding the periodontal defect during surgery. Small bone tips were collected with a bone chisel (Hu‐Friedy) or a bone scraper (MICROSS, Geistlich Pharma AG, Wolhusen, Switzerland), and immediately placed into the defect site until the defect was filled completely. The detached gingival flap was repositioned to its preoperative position and sutured with nylon thread (Softstretch, GC Co., Tokyo, Japan). Teeth with mobility were stabilized using adhesive resin or wire splints, minimizing their impact on healing. All patients were prescribed antibiotics for prophylaxis against postoperative infection and analgesics for pain management after surgery. The sutures were removed 1 week after the procedure. Postoperative care involved suspending oral hygiene for about 1 week, after which regular cleaning was gradually resumed under professional supervision. All surgical procedures were performed by operators who were board‐certified dentists of the Japanese Society of Periodontology (T.M., A.H., I.H., I.M., O.K., O.K., S.S., S.H., H.A., Y.K., Y.T., K.M., T.S., H.S.) ensuring a consistent level of surgical skill and reducing the differences between dentists. All of these dentists were experienced in both FGF‐2 and ABG procedures.

### 2.4 Statistical analysis

The comparison of PPD and BOP positive rates between pre‐ and post‐operation was evaluated using the Wilcoxon signed‐rank test in each group. To compare the clinical outcomes between the FGF‐2 and the ABG groups, analyses were performed based on sex, age, smoking history, presence of systemic disease, preoperative PPD, bone defect morphology, and BDA. Fisher's exact test was used for categorical variables. The Mann–Whitney *U* test was employed to compare continuous variables between the two independent groups. Additionally, an analysis of covariance (ANCOVA) was conducted for VBDIR and PPDI between the two groups, adjusting for potential confounders, including age, sex, smoking history, and the presence of hypertension as covariates.

To explore the association between the clinical effects of FGF‐2 (VBDIR and PPDI) and patient background factors (age, sex, smoking history, presence of systemic disease, preoperative PPD, bone defect morphology, and BDA), multiple regression analysis was conducted. Statistical analyses were performed using SPSS (version 29.0, IBM, Tokyo, Japan) and EZR (Saitama Medical Center, Jichi Medical University, Japan), which is a graphical user interface for R (The R Foundation for Statistical Computing, Vienna, Austria). A *p*‐value of < 0.05 was considered statistically significant. EZR is a GUI for R, and more precisely, it is a modified version of R Commander designed to add statistical functions frequently used in biostatistics.^27^

## 3 RESULTS

### 3.1 Participants

**FIGURE 2:**
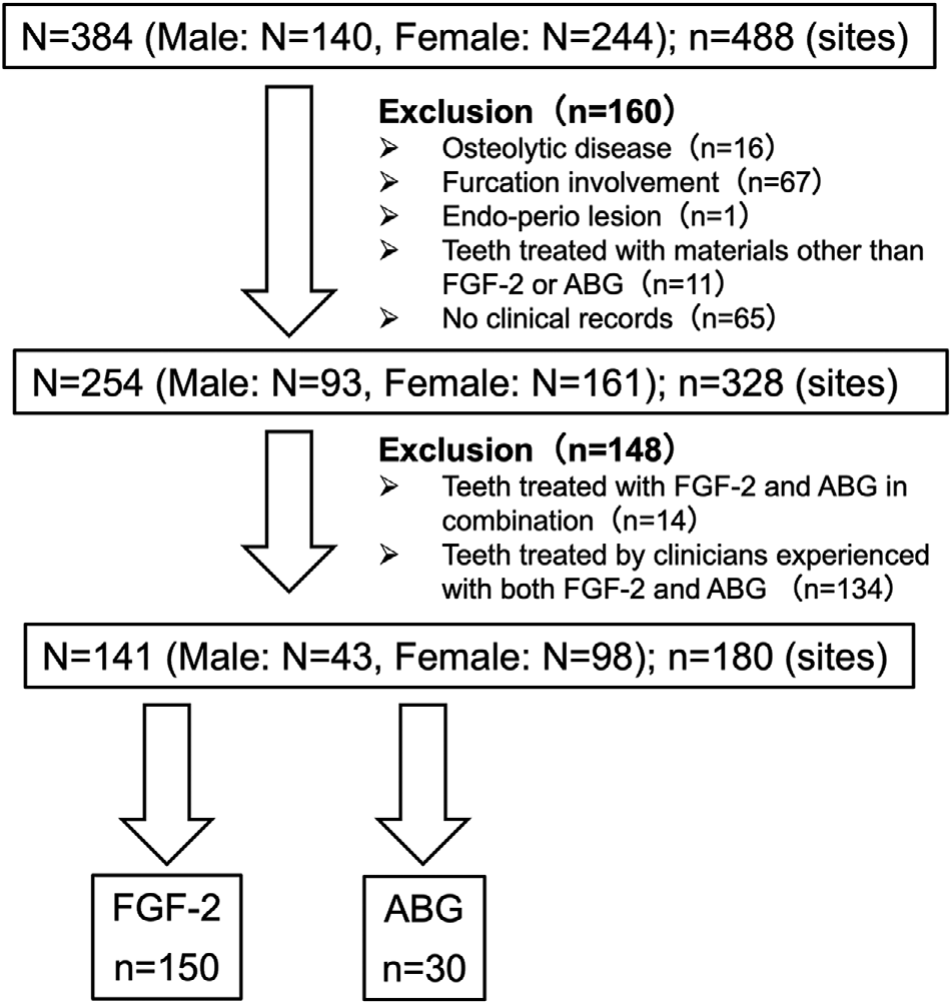
A flowchart illustrating the selection of study participants.

In this study, a total of 488 sites from 384 patients (140 males and 244 females) who met the inclusion criteria were evaluated. Of these, the following sites were excluded: 16 sites with osteolytic diseases, 67 sites with furcation involvement, one site with endo‐periodontal lesions, 11 sites treated with materials other than FGF‐2 or ABG, and 65 sites lacking clinical outcomes or dental radiographs within 2 years before or after surgery. Furthermore, from the remaining 328 sites in 254 patients (93 males and 161 females), 14 sites treated with a combination of FGF‐2 and ABG, as well as 134 sites treated by dentists who performed only one of the two procedures (either FGF‐2 or ABG), were excluded. Ultimately, 180 sites from 141 patients (FGF‐2: 150 sites, ABG: 30 sites) were included in the analysis (Figure 2).

The average age was 53.01 ± 13.63 years. Of the 141 patients, 43 were male and 98 were female (69.5%). A total of 37 patients were smokers (26.2%). There were no significant differences between the FGF‐2 and ABG groups regarding age, sex, or smoking history. Among the systemic diseases associated with periodontal disease, such as hypertension, diabetes, and dyslipidemia, only hypertension had a sufficient sample size for analysis (FGF‐2: 28 sites, ABG: 5 sites). The mean preoperative PPD for all sites was 5.80 ± 1.39 mm, with a bleeding on probing (BOP) positive rate of 71.7%. The average BDA was 36.26 ± 12.27°. Regarding defect morphology, 14 sites (7.8%) were one‐wall defects, 49 sites (27.2%) were two‐wall defects, and 117 sites (65.0%) were three‐wall defects. No significant differences were observed between the FGF‐2 and ABG groups in any of these parameters (Table 1).

**TABLE 1:** Characteristics of patients and periodontal status at baseline.

	All participants	FGF‐2	ABG	*p‐*value^*^
Patient (number)	141	113	28	–
Age (years)	53.01 ± 13.63	52.23 ± 13.91	56.14 ± 11.97	0.16
Sex				
Male	43 (30.5%)	32 (28.3%)	11 (39.3%)	0.26
Female	98 (69.5%)	81 (71.7%)	17 (60.7%)	
Smoke				
Yes	37 (26.2%)	28 (24.8%)	9 (32.1%)	0.47
Systemic disease				
Hypertension	33 (23.4%)	28 (24.8%)	5 (17.9%)	0.62
Diabetes	8 (5.6%)	7 (6.2%)	1 (3.6%)	0.99
Dyslipidemia	4 (2.8%)	3 (2.7%)	1 (3.6%)	0.99
Others	13 (9.2%)	11 (9.7%)	2 (7.1%)	0.99
No. of surgical sites	180	150	30	−
Subjected teeth				
Incisor	35 (19.4%)	30 (20.0%)	5 (16.7%)	0.73
Premolar	77 (42.8%)	62 (41.3%)	15 (50.0%)	
Molar	68 (37.8%)	58 (38.7%)	10 (33.3%)	
Mobility				
0	158 (87.8%)	133 (88.7%)	25 (83.4%)	0.55
1	16 (8.9%)	12 (8.0%)	4 (13.3%)	
2	6 (3.3%)	5 (3.3%)	1 (3.3%)	
PPD (mm)	5.80 ± 1.39	5.81 ± 1.34	5.93 ± 1.21	0.54
BOP				
Positive	129 (71.7%)	104 (69.3%)	25 (83.4%)	0.18
BDA (°)	36.26 ± 12.27	36.62 ± 11.97	35.77 ± 11.88	0.85
Bony wall				
1‐wall	14 (7.8%)	12 (8.0%)	2 (6.7%)	0.47
2‐wall	49 (27.2%)	38 (25.3%)	11 (36.6%)	
3‐wall	117 (65.0%)	100 (66.7%)	17 (56.7%)	

Abbreviations: BDA, bone defect angle; BOP, bleeding on probing; PPD, probing pocket depth.

* Mann‐Whitney *U*‐test or Fisher's exact test.

### 3.2 Periodontal clinical outcomes of FGF‐2 and ABG

**FIGURE 3:**
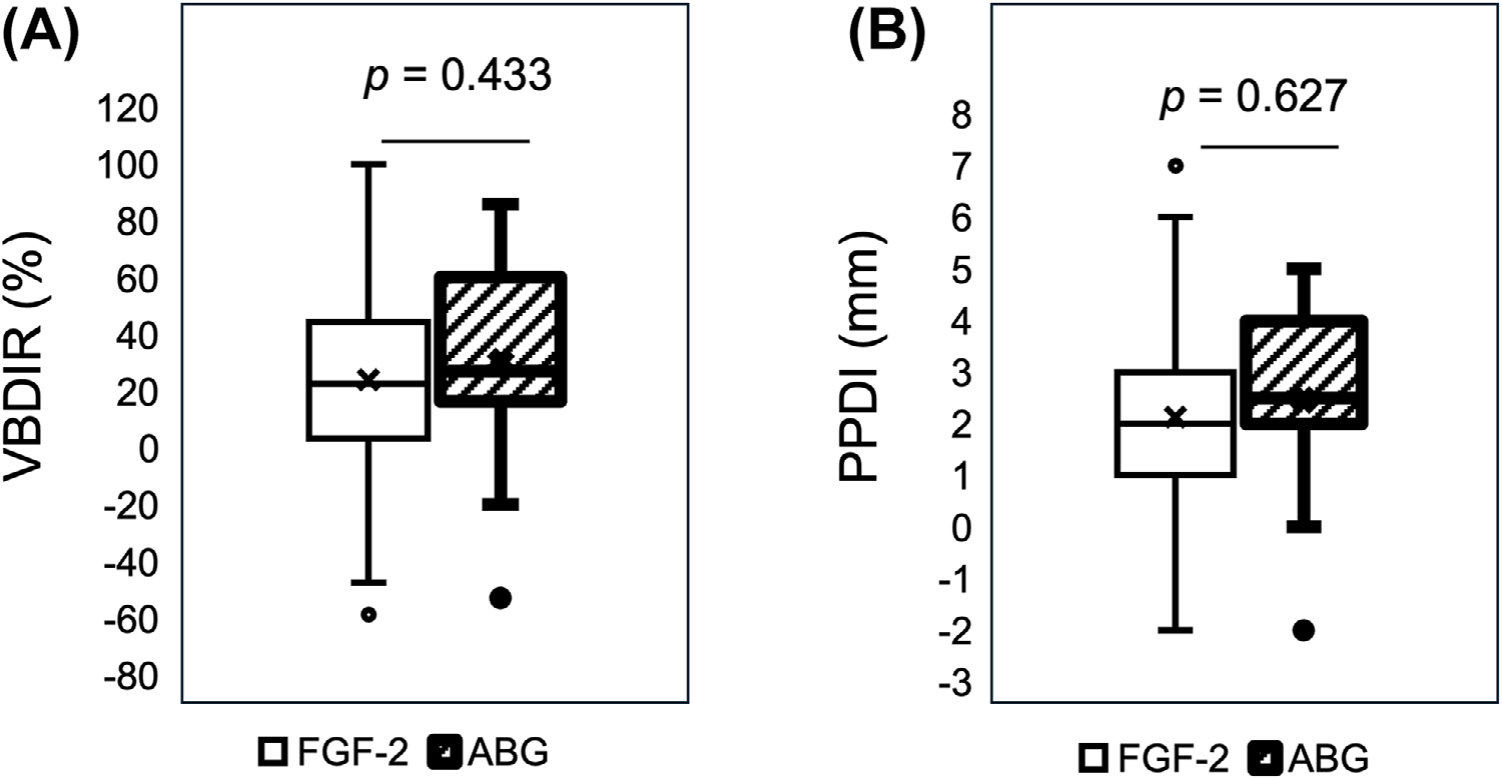
Comparison of VBDIR (A) and PPDI (B) between FGF‐2 and ABG. The *p*‐values are based on the results of analysis of covariance (ANCOVA).

The PPD and BOP positive rates significantly improved postoperatively in both the FGF‐2 and ABG groups (Table 2). The clinical outcomes of FGF‐2 and ABG were compared. The VBDIR was 24.00% ± 30.88% in the FGF‐2 group and 30.53 ± 36.16% in the ABG group. The PPDI was 2.13 ± 1.62 mm for the FGF‐2 group and 2.47 ± 1.43 mm for the ABG group. ANCOVA, adjusted for age, sex, smoking history, presence of hypertension, preoperative PPD, bone defect morphology, and BDA, revealed no statistically significant differences between the two groups in VBDIR (*p* = 0.433) and PPDI (*p* = 0.627) (Figure 3).

**TABLE 2:** Periodontal status at pre‐ and post‐operation.

	Pre‐operation	Post‐operation	*p*‐value*
PPD (mm)			
Total (*n* = 180)	5.80 ± 1.39	3.65 ± 1.31	0.01
FGF‐2 (*n* = 150)	5.81 ± 1.34	3.69 ± 1.29	0.01
ABG (*n* = 30)	5.93 ± 1.21	3.47 ± 1.52	0.01
BOP (%)			
Total	71.7	29.4	0.01
FGF‐2	69.3	28.7	0.01
ABG	83.4	33.3	0.01

BOP, bleeding on probing; PPD, probing pocket depth.

* Wilcoxon signed‐rank test.

### 3.3 Impact of patient factors on clinical outcomes using FGF‐2

The relationship between the clinical outcomes of FGF‐2 and patient‐related factors, including age, sex, smoking history, presence of hypertension, preoperative PPD, bone defect morphology, and BDA, was assessed using multiple regression analysis. For VBDIR, significant associations were positively observed with smoking (*p* = 0.010) and negatively associated with preoperative BDA (*p* = 0.023). For PPDI, a significant negative association was also found with preoperative BDA (*p* = 0.034) (Table 3).

**TABLE 3:** Impact of patients’ factors on clinical efficacy using FGF‐2.

	VBDIR			PPDI		
Parameter	Coef.	95％CI	*p*‐value^*^	Coef.	95％CI	*p*‐value^*^
Age	−0.0022	−0.0060–0.0016	0.26	0.0105	−0.0100–0.0310	0.31
Sex	−0.1099	−0.2291–0.0094	0.07	−0.2250	−0.8629–0.4130	0.49
Smoke	0.1657	0.0408–0.2906	0.01	0.5273	−0.1411–1.1956	0.12
Hypertension	0.0484	−0.0737–0.1705	0.43	0.4582	−0.1948–1.1113	0.17
PPD (mm)	0.0051	−0.0309–0.0410	0.78	0.1598	−0.0326–0.3523	0.10
Bony wall	0.0607	0.0445–0.2906	0.26	0.0858	−0.4766–0.6482	0.76
BDA (°)	−0.0048	−0.0089—0.0007	0.02	−0.0238	−0.0457—0.0019	0.03

Abbreviations: BDA, bone defect angle; CI, confidence interval; Coef., coefficient; PPD, probing pocket depth, PPDI, probing pocket depth improvement (mm); VBDIR, vertical bone defect improvement ratio (%).

* Multiple regression analysis.

## 4 DISCUSSION

Fibroblast growth factor‐2 (FGF‐2) has been reported to significantly promote bone regeneration compared with placebo‐treated sites.^28^, ^29^ FGF‐2 with hyaluronic acid has been reported to significantly improve periodontal wound healing.^30^ On the other hand, autologous bone graft (ABG) has demonstrated benefits in reducing alveolar bone resorption, probing pocket depth (PPD), and improving clinical attachment level (CAL).^31^, ^32^ However, direct comparisons between FGF‐2 and ABG remain limited. Additionally, few studies have investigated the influence of patient background factors such as age, sex, or systemic conditions. Therefore, the present study aimed to compare the clinical outcomes of FGF‐2 and ABG and further to explore how individual characteristics may affect treatment response in cases treated with FGF‐2. To this end, we conducted a retrospective cohort study to evaluate multiple clinical outcomes, despite inherent limitations such as small sample size, variability in follow‐up periods, and incomplete data on intrabony defect characteristics. A total of 180 sites from 141 patients were included in the analysis.

We set the vertical bone defect improvement (VBDIR) and the probing pocket depth improvement (PPDI) as clinical outcomes. The clinical outcomes of FGF‐2 and ABG were initially assessed using ANCOVA, adjusting for age, sex, smoking history, and the presence of hypertension. Both FGF‐2 and ABG led to improvements in vertical bone defect and probing pocket depth compared with preoperative baselines; however, no significant differences were found between the two groups in terms of vertical bone defect improvement rate (VBDIR) and PPD improvement (PPDI). Although ABG has the longest track record in periodontal regenerative therapy, it is associated with limitations such as the invasiveness of autogenous bone harvesting and restrictions on the available quantity. Furthermore, despite its osteoinductive and osteoconductive properties, the regenerative potential of ABG may be compromised if the grafted bone becomes a source of dysbiotic biofilm.^33^ In contrast, FGF‐2 is minimally invasive, easy to apply, and has been reported to promote wound healing and to enhance resistance to dysbiotic biofilm, including the induction of Sharpey's fibers.^34^ These advantages suggest that FGF‐2 may be more beneficial than ABG in periodontal regeneration. While a randomized controlled trial by Kojima et al.^26^ reported significantly superior bone regeneration with FGF‐2 compared with ABG, our study found no statistically significant difference between the two. This discrepancy may be attributed to differences in the study design: our retrospective study had an unequal number of cases between groups and did not exclude patients with systemic conditions such as cardiovascular disease or diabetes, or those with a history of smoking, in contrast to the stricter inclusion criteria of Kojima et al.^26^ The novelty of the present study lies in its examination of clinical efficacy in relation to patient background factors such as age, sex, smoking history, and hypertension.

Multiple regression analysis indicated that smoking history and the preoperative bone defect angle (BDA) may influence the clinical effects of FGF‐2. Interestingly, a smoking history was positively associated with the vertical bone defect improvement rate (VBDIR), a finding that contrasts with previous studies^35^, ^36^, ^37^, ^38^ that reported negative effects of smoking on periodontal treatment outcomes. In the present study, it is possible that some individuals received regenerative therapy despite being advised to stop smoking beforehand. Favorable anatomical features—such as gingival morphology—and good oral hygiene may have contributed to successful regeneration, potentially counteracting the adverse effects of smoking. These results suggest that smoking history may influence the outcome of periodontal regenerative therapy, although this relationship is likely modified by confounding factors. In contrast to previous studies^26^ that excluded smokers, the inclusion of smokers in our analysis highlights the clinical relevance of assessing FGF‐2 efficacy in real‐world settings. However, the relatively small sample size in this study limits the generalizability of the findings. Further investigations with larger cohorts are necessary to confirm these results. Additionally, a negative correlation was observed between preoperative BDA and both VBDIR and PPD improvement (PPDI), indicating that larger defect angles are associated with reduced clinical outcomes. This finding is consistent with previous reports.^39^ Furthermore, one‐wall intrabony defects generally have a poor prognosis in periodontal regenerative therapy. However, our multivariate analysis showed no significant effect of residual bone walls on outcomes, likely due to the predominance of two‐ and three‐wall defects and the small number of one‐wall cases. Subgroup analysis by defect type was avoided to preserve statistical power, so all defects were included. This limitation should be considered when interpreting the findings.

In terms of systemic conditions, hypertension, diabetes, and dyslipidemia were included in the analysis. Among these, only hypertension had a sufficient sample size and was therefore selected for further evaluation. The analysis revealed no significant differences in vertical bone defect improvement rate (VBDIR) or probing pocket depth improvement (PPDI) between hypertensive and non‐hypertensive patients, with both FGF‐2 and ABG demonstrating clinical efficacy in periodontal tissues. Although hypertension is known to impair cellular function through mechanisms such as disrupted bone metabolism, altered remodeling, and increased oxidative stress,^40^ and has been associated with prolonged wound discharge after total hip arthroplasty.^41^ Our findings suggest that well‐controlled blood pressure may not adversely affect the outcomes of periodontal regenerative therapy. The limited number of cases with other systemic conditions, such as diabetes and dyslipidemia, likely reflects the standard clinical practice of performing periodontal surgery only when these underlying conditions are well managed.^42^ Consequently, the number of eligible patients meeting the inclusion criteria for this study was restricted. To more fully assess the influence of systemic health conditions on clinical outcomes, further studies involving broader patient populations will be necessary.

Furthermore, the distinct healing mechanisms of FGF‐2 and ABG may make direct comparison challenging. FGF‐2 functions as a signaling molecule that stimulates angiogenesis, fibroblast proliferation, and the recruitment of periodontal ligament‐derived cells, supporting regeneration of cementum, periodontal ligament, and alveolar bone.^12^ In contrast, ABG primarily provides an osteoconductive scaffold for bone deposition by osteogenic cells,^17^, ^18^ and remains the “gold standard” for alveolar bone augmentation due to the predictable bone fill and low donor‐site morbidity.^19^, ^20^ Histological evidence of true periodontal regeneration with ABG is limited, as most grafts promote bone fill without consistently forming new cementum or ligament.^3^, ^43^, ^44^ Thus, radiographic bone gain may not represent histological regeneration. Overall, FGF‐2 appears more favorable for true periodontal regeneration, while ABG primarily restores volume. Clinically, FGF‐2 may be preferred for attachment reconstruction, whereas ABG may suit wide defects requiring volume stability. In our study, clinical outcomes were comparable, though FGF‐2 was influenced by smoking and BDA, suggesting its proangiogenic^45^ and mesenchymal stem cell‐stimulating effects^12^ may benefit patients with compromised blood supply. Conversely, FGF‐2 may be less suitable for wide‐angled defects where retention is difficult. Combining FGF‐2 with ABG or other scaffolds may enhance periodontal regeneration by complementing each material's healing characteristics, as previous studies have shown improved bone formation and reduced gingival recession.^26^, ^46^, ^47^, ^48^, ^49^ Further prospective studies with histological evaluation are needed to clarify tissue‐level healing dynamics.

This study has several limitations. First, a priori sample size calculation was not performed, and group sizes were imbalanced (FGF‐2: 150 sites; ABG: 30 sites) due to the retrospective design. Patient numbers reflected the availability of eligible cases, which may limit the statistical power. However, including all consecutive cases minimized selection bias and ensured real‐world applicability. The other limitations were the difference in treatment periods: FGF‐2 (REGROTH) became available in 2016, whereas ABG cases were from 2013–2015. Although this temporal gap may have influenced group sizes, treatment protocols, and patient selection criteria remained consistent, and baseline characteristics were comparable, likely minimizing temporal bias. Additionally, the vertical bone defect depth was assessed using dental radiographs; however, standardized radiographic stents were not utilized, which may have introduced measurement inaccuracies. To compensate for this, the vertical bone defect improvement rate (VBDIR) was calculated as a ratio relative to the distance from the cemento‐enamel junction to the root apex, thereby correcting for potential variations and improving measurement reproducibility.^26^ Although cone‐beam computed tomography (CBCT) provides more accurate assessments of bone morphology, dental radiographs were chosen for this study to ensure a sufficiently large sample size, due to their lower radiation exposure, greater accessibility, and ease of use. Furthermore, potential biases, including examination‐ and radiographic‐, and operator‐related biases may have influenced both the radiographic measurements (VBDIR) and clinical probing pocket depth (PPD). To minimize these biases, radiographic evaluations were limited to a small number of examiners, and both intra‐ and inter‐examiner calibrations were conducted to improve measurement consistency. The PPD was measured by board‐certified dentists of the Japanese Society of Periodontology, with the same examiner consistently assessing each patient. However, inter‐examiner calibration for PPD was not performed, and clinical evaluations were not blinded, which may have introduced variability. Moreover, as the operator's surgical technique can impact treatment outcomes, only cases treated by dentists experienced with both FGF‐2 and ABG procedures were included to reduce operator‐related bias. In addition, as all operators were board‐certified dentists of the Japanese Society of Periodontology, the influence of variability in surgical technique was considered minimal. However, this may limit the generalizability of our findings to routine clinical practice. As the other major limitations, there was the absence of CAL data. Although the clinical attachment level is a key outcome measure, it was not consistently recorded due to the retrospective study design and its susceptibility to variability from gingival recession and difficulty in locating the cement‐enamel junction. Instead, PPD reduction and radiographic bone fill were used as more reliable and consistently available measures. Finally, this study did not include a control group that received no regenerative intervention. Ideally, to determine the true regenerative effect, a comparison group undergoing only gingival flap debridement would be necessary. However, since regenerative therapy has become the standard of care for vertical bone defects, assembling a sufficiently large non‐treatment control group is challenging. Therefore, this study focused on comparing FGF‐2, a novel regenerative agent, with ABG, historically considered as a reliable material in periodontal regenerative therapy.

## 5 CONCLUSION

This retrospective cohort study demonstrated that fibroblast growth factor‐2 (FGF‐2) and autologous bone grafting (ABG) both contributed to clinical improvements in periodontal parameters, suggesting their potential effectiveness in periodontal tissue regeneration. Although the regenerative potential of FGF‐2 is well established, our study provides new clinical insight by directly comparing it with ABG and by identifying patient‐related factors, such as smoking and bone defect angle, that may guide therapy selection.

## AUTHOR CONTRIBUTIONS

**Hidetaka Ideguchi**: Developed the concept for this study. **Kazuhiro Omori**: Developed the concept for this study. **Shogo Takashiba**: Developed the concept for this study; reviewed the manuscript. **Toshiki Matsumoto**: Provided substantial assistance in data correction; performed statistical analyses. **Mai Sakamoto**: Provided substantial assistance in data correction. **Takayuki Ishii**: Provided substantial assistance in data correction. **Yasuki Nonomura**: Provided substantial assistance in data correction. **Shin Nakamura**: Performed statistical analyses; wrote the initial draft of the manuscript. **Kazu Takeuchi‐Hatanaka**: Performed statistical analyses; wrote the initial draft of the manuscript; reviewed the manuscript. **Keisuke Okubo**: Wrote the initial draft of the manuscript. **Yuki Ito‐Shinoda**: Wrote the initial draft of the manuscript. **Tadashi Yamamoto**: Reviewed the manuscript. All authors approved the final version of the manuscript.

## CONFLICT OF INTEREST STATEMENT

The authors declare no conflicts of interest related to this study.
